# The prevalence of hearing loss in children and adolescents with cancer

**DOI:** 10.1016/S1808-8694(15)30120-8

**Published:** 2015-10-19

**Authors:** Aline Medeiros da Silva, Maria do Rosário Dias de Oliveira Latorre, Lilian Maria Cristofani, Vicente Odone Filho

**Affiliations:** a M.S. Student in Public Health - USP School of Public Health. Speech and Hearing Therapist; b Associate Professor - School of Public Health - USP, Full Professor- Department of Epidemiology - School of Public Heath - University of São Paulo (USP); c PhD in Pediatrics - University of São Paulo Medical School. Assistant Physician - Department of Pediatric Cancer - ITACI. University of São Paulo Hospital; d Associate Professor - USP Medical School. Head of the Pediatric Cancer Institute- ITACI. University of São Paulo Hospital. School of Public Health - University of São Paulo (USP)

**Keywords:** childhood cancer, ototoxicity, hearing loss, chemotherapy

## Abstract

The treatment of cancer in children has several side effects, including ototoxicity. Inner ear structures may be affected and hearing loss may ensue. **Aim:** To estimate the prevalence of hearing loss in patients with cancer using the American Speech-Language-Hearing Association (ASHA), the Pediatric Oncology Group Toxicity (POGT), and the Bilateral Hearing Loss (PAB) criteria. Study design: a prospective study. **Material and Methods:** 94 patients admitted between 2003 and 2004 were analyzed. Visual inspection of the external auditory meatus and an audiologic evaluation were done. Descriptive statistics was used to characterize the sample, and Kappa statistics was used to investigate concordance of hearing loss in the three types of classification. **Results:** The prevalence of hearing loss was 42.5% using ASHA, 40.4% using POGT, and 12.8% using PAB. The concordance of hearing loss was weak for POGT and PAB (k=0.36) and for PAB and ASHA (k=0.33). The concordance between ASHA and POGT was almost perfect (k=0.96). **Conclusions:** Hearing loss is an important side effect of the treatment of cancer in children. Periodic audiology monitoring is recommended to detect early hearing loss and to revise the treatment if necessary. Adoption of a classification system that detects mild hearing loss (ASHA) is recommended.

## INTRODUCTION

The increase in survival rates of children and adolescents with malignant tumors in the last two decades reflects more effective treatments - including combined chemotherapy, better diagnostics, better surgical techniques and radiotherapy, the combined use of different treatment modalities, better support and increase in survival and quality of life^1^, ^2^, ^3^. Nonetheless, children and teenagers with cancer are exposed to the most diverse sort of side effects, especially when submitted to chemotherapy, which despite being one of the most promising means to fight cancer, depending on the chemotherapeutic agents employed it can cause undesirable side effects.

Side effects may manifest themselves earlier on, or in the long run, depending on treatment type and child’s age. Hearing loss is among these many side effects^4^, ^5^.

Ototoxicity is defined as a toxic reaction that affects the inner ear, both the auditory and/or the vestibular system and may cause hearing loss^6^. Ototoxicity has taken an important role, especially in younger children, having seen that it is usually irreversible and, thus, it means a loss in the long run^7^, ^8^.

One commonly used classification to assess hearing losses in audiology clinics is the one from Davis and Silverman (1970), mentioned by Russo and Santos (1993)^9^. It is based on determining the degree of hearing loss from air conduction threshold average in the frequencies of 500, 1000 and 2000 Hz - the ones most important for speech, considering normal hearing until 25 dBHL (decibel hearing level). However, this classification is the same for adults and children, regardless of the disease, and today we also consider the frequency of 4,000 Hz as being important.

According to Haggard and Primus (1999)^10^, the scales used to classify hearing loss are not similar, and such fact makes it difficult to compare prevalence. There is much disagreement on which would be the most adequate classification to use for hearing loss, however the consensus is that it is important to adopt a differentiated hearing loss classification for children. According to Northern and Downs (1989)^11^, the ideal tonal threshold for a child is 15 dB or less in all audiogram frequencies (from 250 to 8000 Hz), differently from adults, in which the mean value between 20-25 dB is acceptable.

In assessing the child’s hearing loss, it is important to highlight that even mild loss, which would not impact adults, may interfere in children’s capacity to acquire and develop language skills. Hearing loss, even if transitional, between 25 and 35 dBHL, this reduction is not enough to prevent the child from hearing, however, it may impair the child’s ability to understand some phonema^11^.

In 1991, the American Speech-Language-Hearing Association - ASHA (Hersh and Johnson, 2003)^12^ proposed a hearing loss classification for children. Hearing loss is determined according to the different degrees of difficulty for communication, considering normal hearing the one between 10 and 15 dBHL, mild hearing loss between 16 and 25 dBHL, light hearing loss between 26 and 40 dBHL, moderate hearing loss between 41 and 55 dBHL, moderately severe hearing loss between 56 and 70 dBHL, severe hearing loss between 71 and 90 dBHL and profound hearing loss those above 90 dBHL.

In 1994, the American Speech-Language-Hearing Association - ASHA proposed another specific classification for patients treated with ototoxic drugs, the Ototoxicity Criteria (ASHA, 1994)^13^. Such classification classifies hearing loss by means of comparing the results of previous audiologic evaluations, in A (threshold increase in 20 dB or more in one frequency), B (threshold increase in 10 dB or more in two sequential frequencies) and C (no response in three sequential frequencies, which were present in prior audiologic evaluations).

According to Brock et al. (1991)^8^, hearing loss specifically induced by cisplatin has been measured by pre and post treatment audiograms. Notwithstanding, the existing classifications for hearing loss are not adequate to be used in children with cancer, since the most common classifications usually determine the degree of hearing loss based on the mean value of air conduction in the frequencies of 500, 1000 and 2000 Hz (as the classification from Davis and Silverman, 1970). Thus, the authors proposed an exclusive classification for children treated with cisplatin or other ototoxic drugs. In such classification, hearing loss is classified in levels, according to the frequency that presented tonal threshold below 40 dBHL: Level 0 (hearing thresholds below 40 dBHL in all the frequencies), Level 1 (hearing loss above or equal to 40 dBHL in 8000 Hz), Level 2 (Hearing loss above or equal to 40 dBHL in 4000 Hz and below), Level 3 (hearing loss above or equal to 40 dBHL in 2000 Hz and below) and Level 4 (hearing loss above or equal to 40 dBHL in 1000 Hz and in lower frequencies). The classification proposed considers that hearing loss above or equal to 40 dBHL, in any frequency, implies loss in speech recognition and, the more it affects speech frequencies, the more harm it brings. The authors consider that after 40 dBHL the hearing loss was surely caused by an external agent, the ototoxic drug in this case - and that in lower intensities, the hearing loss could happen due to other agents.

In 1999, the Pediatric Oncology Group Toxicity - POGT (Huang et al., 2002)^14^ developed a hearing loss classification for children treated with chemotherapeutic agents. This classification also classifies the loss in levels: Level 0 (normal hearing), Level 1 (hearing loss between 20 and 40 dBHL in frequencies above 4000 Hz), Level 2 (hearing loss above or equal to 40 dBHL in frequencies above or equal to 4000 Hz), Level 3 (hearing loss above or equal to 40 dBHL in frequencies above 2000 Hz) and Level 4 (hearing loss above or equal to 40 dBHL in 2000 Hz and below). This classification considers the hearing loss above or equal to 20 dBHL in the frequencies above 4000 Hz already implies in speech recognition loss and that, the more it affects frequencies between 2000 Hz and below, the greater is the communication difficulty.

Another classification was proposed by the National Cancer Institute (NCI), the Common Toxicity Criteria version 2.0 (CTC) (1999)^15^. This classification classifies hearing loss in levels from 0 to 4, according to the symptoms and the results from the audiologic evaluation: Level 0 (normal hearing), Level 1 (mild hearing loss, no complaints), Level 2 (tinnitus or hearing loss that does not require the use of a hearing aid), Level 3 (tinnitus or hearing loss that requires the use of a hearing aid), Level 4 (severe unilateral or bilateral hearing loss). This classification is subjective - it is based on clinical complaints and on the judgment regarding the relevance or not of using a hearing aid - issues which are difficult to assess, especially in small children.

Recently, another classification was proposed by the National Cancer Institute (NCI), the Common Terminology Criteria for Adverse Events version 3.0 (CTCAEv3) (2003)16. This classification breaks down hearing loss in levels from 1 to 4, by comparing the results of prior audiologic assessments: Level 1 (threshold increase between 15 and 25 dB in relation to the initial audiologic exam, in two or more sequential frequencies in at least one ear), Level 2 (threshold increase between 25 and 90 dB in two sequential frequencies), Level 3 (threshold loss greater than or equal to 20 dB bilaterally in speech frequencies, or greater than or equal to 30 dB unilaterally) and Level 4 (hearing loss requiring a hearing aid or even a cochlear implant).

Although the classifications proposed by Brock et al. (1991)^8^ and by the Pediatric Oncology Group Toxicity (POGT)14 were made in order to assess ototoxicity in specific situations, all of them used the same auditory thresholds in accordance with intensity in different frequencies, which can be used independently from the assessment carried out prior to treatment. In the literature we did not find any study that compared and discussed the relevance of using these classifications. Having said that, the goals of the present investigations are to determine hearing loss prevalence among children and teenagers with cancer using the classifications from the American Speech-Language-Hearing Association (ASHA), of Bilateral Hearing Loss (BHL) and from the Pediatric Oncology Group Toxicity (POGT), and check the agreement of these three classifications in the diagnosis of hearing loss.

## MATERIALS AND METHODS

The present investigation was developed in an institute which is the national reference center for the treatment of pediatric cancer, and was approved by the Research Ethics Committee of the School of Public Health of the University of São Paulo, under protocol # 1186 and the Ethics Committee for the Analysis of Research Projects - CAPPesq of the University of São Paulo Hospital Executive Board, protocol # 104/06.

After surveying patients’ charts, we noticed that 325 new cases were seen in 2003; and 342 in 2004; most of them, 469 children (= 70.3%), did not have the diagnosis of cancer confirmed and were referred to another unit for specific treatment.

Therefore, we selected 87 new cases seen in 2003 and 111 new cases seen in 2004 for our investigation, making up a total of 198 patients. Of these, 44 (22.2%) died and 12 (6.1%) were transferred to other treatment centers located in other cities/capitals, leaving a total of 142 patients. Since pre-treatment audiologic evaluation is not carried out routinely in this institute, these patients were not included in this study.

We first assessed the medical chart of these patients in order to fill out the form and, later we scheduled their audiologic exam.

Among the 142 patients, we were able to do the audiologic exam in 94 patients.

### The audiologic exam

We first carried out an anamnesis in order to look for any symptom or hearing loss complaint. Later on, we visually inspected the external acoustic meatus with an otoscope, checking for wax or any other thing that could prevent these exams. If a wax ball was seen, the patient was then referred to the otorhinolaryngologist to remove it, and only afterwards the exam was carried out.

Following that, auditory thresholds were evaluated, by means of conditioned response methods (audiometry with visual reinforcement - children of up to two years of age; conditioned audiometry within a ludic activity - children between 2 and 5 years; or tonal threshold audiometry - children above 5 years, with the aim of determining their hearing thresholds. This test was carried out in an acoustic booth, using the Maico MA-41 audiometer with TDH-39 phones, duly calibrated according to current standards, in the frequencies of 250 to 8000 Hz and, by bone conduction, in the frequencies of 500 to 4000 Hz, whenever necessary in order to confirm the findings. Later on, we carried out tympanometry and acoustic reflexes test using the Danplex ZA-28 impedance meter device. All the tests were carried out by the same audiologist (AMS).

In order to define the hearing loss, three classifications were used: American Speech-Language-Hearing Association (ASHA), Bilateral Hearing Loss (BHL) and the Pediatric Oncology Group Toxicity (POGT).

The sample was characterized by means of descriptive statistics (mean, standard deviation, median and proportions) and we analyzed the three classifications insofar as their agreement is concerned in terms of hearing loss by means of the Kappa statistics. In all statistical analysis we used the 5% level of statistical significance.

We used the Epi Info v. 6.04 for DOS software for database double entry and data consistence; and the SPSS v. 12.0 for Windows for statistical analyses.

The audiologic evaluation did not involve any invasive procedure and all parents and guardians signed the informed consent. At the end of the exam, we handed the results of this audiologic evaluation to the children’s parents or guardians.

## RESULTS

There were more males in the sample (52.1%) than females. Caucasians were also more common (83.0%), followed by browns (11.7%). Age at diagnosis varied between 0 and 18 years (mean of 6.8 years; standard deviation of 4.9 years; median of 5.6 years), and less than half of the patients were diagnosed before 5 years of age (45.7%). Current age varied between 1 and 18 years (mean age of 8.6 years; standard deviation of 4.8 years; median of 7.4 years) and the most frequently found age range was between 5 and 9 years (38.3%), followed by that of 10 years and above (36.2%).

The most frequent diagnosis was of lymphoid leukemia (30.8%), followed by the bone tumors group (8.5%) and Willms’s tumor (8.5%) (Table 1). Patients diagnosed with lymphoid leukemia, bone tumors, neuroblastoma, meduloblastoma, adrenal carcinoma, nasopharyngeal carcinoma, hepatoblastoma, soft tissue sarcoma, Hodgkin lymphoma, female-germ cells’ tumor, male-germ cells’ tumor and peripheral neuroectodermal tumor patients used cisplatin and/or ifosfamide. Patients diagnosed with Willms’s tumor, retinoblastoma, non-lymphoid acute leukemia, malignant histiocytosis, CNS tumor, non-Hodgkin lymphoma and thyroid carcinoma did not use these drugs.

**Table 1:** Number and percentage of patients, according to topography and the use of cisplatin and/or ifosfamide.

Topography	#	%
Lymphoid leukemia	29	30.8
Bone tumors (osteossarcoma, Ewing’s sarcoma)	8	8.5
Willms’s tumor	8	8.5
Retinoblastoma	7	7.4
Neuroblastoma	6	6.3
Meduloblastoma	5	5.3
Adrenal carcinoma	5	5.3
Nasopharyngeal carcinoma	4	4.3
Hepatoblastoma	4	4.3
Soft tissue sarcomas (rabdomyossarcoma, fuso-cellular)	4	4.3
Non-lymphoid acute leukemia	4	4.3
Hodgkin’s lymphoma	2	2.1
Malignant histiocytosis	2	2.1
Stem cells tumor	2	2.1
CNS tumor (glioblastoma multiforme)	1	1.1
Non-Hodgkin lymphoma	1	1.1
Thyroid carcinoma	1	1.1
Peripheral neuroectodermal tumor	1	1.1

Total	94	100.0

(used by one patient who relapsed)

Of the 94 patients, 38 (40.4%) had pain in their bodies at the time of diagnosis, followed by fever (34.0%) and increase in body mass volume (31.9%). The less frequent symptom was motor change (3.2%), mentioned only by three patients.

Among the 94 patients, 67 (71.3%) were alive without chemical or radiotherapy treatment at the time of the last visit recorded in their medical charts; and 27 (28.7%) were alive and under treatment.

Table 2 describes the use of medication with these patients. 21 patients (22.3%) used Cisplatin - considered highly ototoxic, at a mean individual dose of 78.09 mg/m^2^ (standard deviation = 32.69 mg/m^2^), maximum individual dose of 140 mg/m^2^, maximum cumulative dose of 1120 mg/m^2^ and average number of treatment cycles of 4.24 (standard deviation = 1.55 cycles).

**Table 2:** Number and percentage of patients according to drug used.

Drug	#	%*
Folinic acid	31	33.0
Dactinomycin	18	19.1
Adriamycin	39	41.5
Arabinosil Citosine (high dose)	15	16.0
Arabinosil Citosine (low dose)	33	35.1
L-Asparaginase (U)	26	27.7
Carboplatin	21	22.3
Cyclophosphamide	46	48.9
Cisplatin	21	22.3
Daunorubicin	27	28.7
Dexametasone	35	37.2
Etoposide	42	44.7
5-Fluorouracil	7	7.4
Ifosfamide	20	21.3
6-Mercaptopurin	32	34.0
Metotrexate (high dose)	34	36.2
Metotrexate (low dose)	35	37.2
Prednisone	5	5.3
Teniposide	11	11.7
Topotecan	6	6.4
Vancomycin	6	6.4
Vinblastine	4	4.3
Vincristine	55	58.5
Metotrexate (MADIT)	29	30.9
AraC (MADIT)	29	30.9
Dexametasone (MADIT)	24	25.5

* Percentage calculated in relation to the 94 patients.

21 patients (22.3%) received carboplatin, cisplatinanalogue drug, however of lower ototoxic potential, at a mean individual dose of 330.75 mg/m^2^ (standard deviation = 208.21 mg/m^2^), maximum individual dose of 775 mg/m^2^, maximum cumulative dose of 4500 mg/m^2^ and number of cycles mean value of 4.14 (standard deviation = 2.82 cycles).

Most commonly mentioned hearing complaint was otitis (22.3%), followed by otalgia (16.0%), dizziness (16.0%), hearing difficulties (14.9%) and upper airway infections (13.8%).

Table 3 shows the hearing loss classification according to the three different classifications. We see that according to the ASHA’s classification, 54 (57.5%) patients had hearing thresholds within normal limits and 40 (42.5%) had some degree of hearing loss. Considering BHL, 82 (87.2%) patients had hearing thresholds classified in Level 0 and 12 (12.8%) patients with some degree of hearing loss. And finally, considering POGT’s classification, only 56 (59.6%) patients under levels 0 and 38 (40.4%) had some degree of hearing loss, in other words, result very similar to the one found by using the ASHA classification.

**Table 3:** Number and percentage of patients according to hearing loss classification.

Classification	Category	Nº	%
ASHA	Normal	54	57,5
	Mild loss	16	17,0
	Light loss	14	14,9
	Moderate loss	2	2,1
	Moderately severe loss	7	7,4
	Severe loss	1	1,1
BHL	Level 0	82	87,2
	Level 1	5	5,3
	Level 2	2	2,1
	Level 3	4	4,3
	Level 4	1	1,1
POGT	Level 0	56	59,6
	Level 1	29	30,8
	Level 2	3	3,2
	Level 3	5	5,3
	Level 4	1	1,1

	TOTAL	94	100,0

The agreement in the hearing loss diagnosis according to classifications POGT and PAB was weak (Kappa = 0.36; p <0.001; Table 4), and the same thing happened for BHL in relation to ASHA’s (Kappa = 0.33; p<0.001; Table 5). The agreement between ASHA and POGT was almost perfect (Kappa = 0.96; p<0.001; Table 6).

**Table 4:** Number and percentage of patients according to the agreement between classification methods BHL and POGT.

	POGT					
BHL	Level 0		Levels 1 through 4		Total	
	Nº	%	Nº	%	Nº	%
Level 0	56	100,0	26	68,4	82	87,2
Levels 1 a 4	--	--	12	31,6	12	12,8

TOTAL	56	100,0	38	100,0	94	100,0

Kappa = 0,36 (p<0,001)

**Table 5:** Number and percentage of patients according to the agreement between classification methods BHL and ASHA.

	ASHA					
BHL	Normal		Loss		Total	
	Nº	%	Nº	%	Nº	%
Level 0	54	100,0	28	70,0	82	87,2
Levels 1 through 4	--	--	12	30,0	12	12,8

TOTAL	54	100,0	40	100,0	94	100,0

Kappa = 0,33 (p<0,001)

**Table 6:** Number and percentage of patients according to the agreement between classification methods POGT and ASHA.

	ASHA					
BHL	Normal		Loss		Total	
	Nº	%	Nº	%	Nº	%
Level 0	54	100,0	2	5,0	56	59,6
Levels 1 through 4	--	--	38	95,0	38	40,4

TOTAL	54	100,0	40	100,0	94	100,0

Kappa = 0,96 (p<0,001)

## DISCUSSION

One of the first things to be highlighted is the hearing evaluation itself is that the institute where this study was conducted does not have a department of audiology, and is also not specialized in speech and hearing therapy. This is not a particularity of this institute, having seen that in Brazil pediatric cancer treatment centers still do not have a common practice of monitoring patients’ hearing. Thus, we recommend that cancer treatment authorities should be concerned in installing audiology services in cancer treatment centers in order to follow these children and teenagers with cancer and also those with other diseases that may have their hearing compromised by treatment.

In the hearing loss prevalence analysis by the ASHA classification, we found 42.5% of the patients with mild to severe hearing loss. In the literature we did not find any study that used the ASHA classification. It is important to stress that this classification is the strictest among the classifications adopted. Patients who had hearing threshold above 15 dBHL in any frequency were analyzed as having hearing loss. This classification was very much in agreement with the POGT classification (Kappa=0.96), and the only difference was that two patients were classified as Level 0 by POGT, but were considered as having mild loss in frequencies below 4000 Hz by the ASHA classification.

The major agreement in the hearing loss diagnosis between ASHA’s and POGT’s classification happened thanks to the threshold used as cutting point to determine the hearing loss (15 dBHL for ASHA and 20 dBHL for POGT).

When the POGT’s classification was used to classify hearing losses, 40.4% of the patients evaluated had hearing loss between levels 1 and 4. Among the studies that used this classification, the one from Marina et al. (2005)17 with 24 patients with germ cells tumors, treated with cisplatin, found 75% of patients with hearing loss between levels 2 and 4. In the study developed by Ruiz et al. (1989)18, the researchers used the threshold above or equal to 20 dBHL to classify the hearing loss, and 100% of their patients treated with cisplatin had hearing loss.

In the BHL, only those thresholds above or equal to 40 dBHL are classified as hearing loss. Thus, the number of patients with hearing loss using this classification is much lower when compared to the other two. The agreement between the BHL and the ASHA classifications was weak (Kappa=0.33), and the same happened with the POGT classification (Kappa=0.36).

When we used the BHL classification, 12.8% of the patients evaluated had hearing loss between levels 1 and 4. Since this classification only considers hearing loss above 40 dBHL, the mild or light hearing loss in the ASHA classification is classified as Level 0 in the BHL.

In Skinner et al.’s study (1990)7 involving 22 children and adolescents diagnosed with solid tumors and treated with cisplatin, 73% had hearing loss. Brock et al. (1991)8 found hearing loss in 48% of the 29 children with different diagnoses, treated with cisplatin.

Other studies adopted a threshold equal to or above 25 dBHL in any frequency, between 250 and 8000 Hz as a means of hearing loss classification. The percentage of loss varied between 43% and 81%^2^, ^19^, ^20^, ^21^.

Allen et al. (1998)20 carried out a retrospective study with 11 children below 18 years of age who received cisplatin during chemotherapy between 1985 and 1994. They found hearing loss between 25 and 90 dBHL in 81% of the children. We found similar figures in the present investigation (83%), when compared to the POGT classification (the closest one for the cutting point at 25 dBHL).

Another means to analyze and classify hearing loss was by comparing the hearing thresholds of the audiologic evaluations before and after treatment. Studies which adopted an increase in the threshold in any frequency equal to or greater than 10 dBHL when compared to the previous audiologic evaluation, found between 77% and 88% of hearing loss in the patients evaluated^22^, ^23^. In the study carried out by Berg et al. (1999)^24^, hearing loss happened to only 26% of the children assessed.

Other studies did not report on which was the classification used to classify hearing loss, and hearing loss rate varied between 7% and 42%^25^, ^26^, ^27^.

Therefore, we see that comparing hearing loss in children and teenagers with cancer is very difficult - above all - by the use of more or less strict criteria to assess hearing.

Smits et al. (2006)^28^ stressed that hearing loss, even when mild, if not detected early on may cause problems in the long run. Hearing loss diagnoses in patients being treated for cancer is of paramount importance; since it may preserve their communications capacity and avoid that when they recover from cancer they may have hearing sequelae because of their prior treatment^29^.

We must highlight that using more strict criteria, such as the one proposed by the American Speech-Language-Hearing Association - ASHA, for hearing loss in children is fundamental, because in order to communicate well, the child must be able to hear, identify and discriminate all speech-related sounds. A reduction in hearing, even if mild, compromises the development of such skills, impairing understanding and thus, oral communication, it may cause emotional, behavioral and educational problems.
